# 

**Published:** 1902-02-15

**Authors:** 


### NOTICE TO CORRESPONDENTS.

All MSS., Letters, Books for Review, and other matters intended for the Editor should be addressed The Editor, "The Hospital," The Hospital Building, 28 & 29 Southampton Street, Strand, W.C.

The Editor cannot undertake to return rejected MS., even when accompanied by stamped directed envelope.

**Notice to Advertisers and Subscribers.**

All Advertisements, Orders for Copies of The Hospital, and Business Communications should be addressed to **THE MANAGER (Not to the Editor),** The Hospital, 28 & 29 Southampton Street, Strand, London, W.C.

The Cost of Subscription, post free, is as follows:—Per week, 2½d.; per quarter, 2s. 8d.; per half-year, 5s. 3d.; per annum, 10s. 6d. Foreign:—Per annum, 15s. 2d., payable, in all cases, in advance.

**NOTICE.—Vol. XXX. of "The Hospital" (April 1901, to September 1901), in handsome cloth binding can now be obtained at the office of this Journal, price, 7s. 6d. Cases for binding the Numbers contained in this Volume 1s. 6d. Index to Vol. XXX., 2½d., post free.**

**The Monthly Part for February is now ready. Price 6d., by post 8d.**

### BOOKS RECEIVED.

T. B. Browne, Limited.

"The Advertiser's A B C."

Swan Sonnenschein and Co.

"Recent Object Lessons in Penal Science." By A. R. Whiteway, M.A.

Rebman, Limited.

"The Medical Treatment of Gall-stones." By J. H. Reay, M.A., M.D.

Hickson, Ward and Co.

"The Kew Cowl Tests." Preface by Perry Fairfax Nursey, C.E.

John Wright and Co.

"'First Aid' to the Injured and Sick: an Advanced Ambulance Handbook." By F. J. Warwick, B.A., M.B., M.R.C.S., L.S.A., and A. C. Tunstall, M.D., F.R.C.S., Ed.

Smith, Elder and Co.

"Artificial Feeding and Food Disorders of Infants." By W. B. Cheadle, M.A., M.D., F.R.C.P. Revised by F. J. Poynton, M.D., M.R.C.P.

Henry Renshaw.

"A Manual for Assistants' Examination, Apothecaries' Hall." By Mabel F. Stanley.

### THE STUDENT OF MEDICINE.

### APPOINTMENTS.

**William C. Allardyce**, M.D.Glasg., appointed Assistant Surgeon to the North Staffordshire Infirmary and Eye Hospital. **T. W. S. Browne**, M.R.C.S., L.R.C.P.Lond., appointed Certifying Factory Surgeon for the Chatham District. **T. T. Brunyate**, M.A., M.D.Oxon., appointed Physician to St. Bartholomew's Hospital, Rochester. **Howard Cane**, M.D., L.R.C.P.Lond., M.R.C.S.Eng., appointed by the London County Council Medical Officer to the Staff of the Crossness Outfall Works. **G. M. Edwards**, M.B., B.C.Cantab., appointed Certifying Factory Surgeon for the Harrow District of Middlesex. **S. Elliot**, M.D.Edin., appointed Certifying Factory Surgeon for the Wick District of Caithness. **J. Lacey Firth**, M.D., M.S.Lond., F.R.C.S.Eng., appointed Surgeon to the Bristol General Hospital. **John R. Harper**, M.R.C.S., L.R.C.P.Lond., appointed Medical Officer of Health to the Barnstaple Urban District. **V. Howard**, M.R.C.S., L.R.C.P.Lond., appointed Medical Officer of Health for the Borough of Buckingham. **Henry Greville Kyle**, B.A., M.B., B.Ch.Oxon., appointed Assistant Surgeon to the Bristol General Hospital. **J. H. Meachan**, M.R.C.S., L.R.C.P.Lond., appointed Medical Officer of Health for the Wadebridge Urban District. **David J. Roberts**, M.B., Ch.B.Edin., appointed Medical Superintendent of the Westmorland Sanatorium for Consumption, Meathop. **S. P. Sunderland**, M.D.Brux., M.R.C.P.Lond., appointed Physician-in-Chief to the Royal Maternity Charity. **H. J. Waring**, M.S., B.Sc., F.R.C.S., appointed an Assistant Surgeon to St. Bartholomew's Hospital. **James Wheatley**, M.D.Lond., D.P.H., appointed Medical Officer of Health for the County of Shropshire. **Robert Arthur Young**, M.D., B.Sc.Lond., M.R.C.P.Lond., appointed Assistant Physician to the Middlesex Hospital.

St. Bartholomew's Hospital.—The following appointments have been made: House-Physicians.—**H. L. P. Hulbert**, M.A.Cantab., M.R.C.S., L.R.C.P., **R. H. Winick**, M.A., M.B., B.C.Cantab.; **A. E. Thomas**, M.B.Lond., M.R.C.S., L.R.C.P.; **J. Stirling Hamilton**, B.A.Cantab., M.R.C.S., L.R.C.P.; **H. H. Weir**, B.A.Cantab., M.R.C.S., L.R.C.P. Intern, April, 1902.—**H. G. Pinker**, M.R.C.S., L.R.C.P. Extern, April, 1902.—**F. C. Shrubshall**, M.A., M.B., B.Sc.Cantab. Extern, July, 1902.—**R. T. Worthington**, M.R.C.S., L.R.C.P.

